# Use of Serious Games in Interventions of Executive Functions in Neurodiverse Children: Systematic Review

**DOI:** 10.2196/59053

**Published:** 2024-12-18

**Authors:** Luis Carlos Rodríguez Timaná, Javier Ferney Castillo García, Teodiano Bastos Filho, Alvaro Alexander Ocampo González, Nazly Rocio Hincapié Monsalve, Nicolas Jacobo Valencia Jimenez

**Affiliations:** 1 Faculty of Engineering Universidad Santiago de Cali Santiago de Cali Colombia; 2 Faculty of Engineering Universidad Autónoma de Occidente Santiago de Cali Colombia; 3 Faculty of Engineering Universidade Federal do Espírito Santo Vitória Brazil; 4 Faculty of Health Universidad Santiago de Cali Santiago de Cali Colombia

**Keywords:** executive functions, neurodiversity, serious games, cognitive training, therapeutic interventions

## Abstract

**Background:**

Serious games (SG) have emerged as promising tools for cognitive training and therapeutic interventions, especially for enhancing executive functions. These games have demonstrated the potential to support individuals with diverse health conditions, including neurodevelopmental and cognitive disorders, through engaging and interactive experiences. However, a comprehensive understanding of the effectiveness of SG in enhancing executive functions is needed.

**Objective:**

This systematic review aims to assess the impact of serious games on executive functions (EF), focusing on attention, working memory, cognitive flexibility, and inhibitory control. In addition, it explores the integration of SG into educational and therapeutic settings for individuals with cognitive and neurodevelopmental conditions. Only open access articles published from 2019 to the search date were included to capture the most recent advancements in the field.

**Methods:**

A comprehensive search was conducted on June 20, 2024, across Scopus, Web of Science, and PubMed databases. Due to limited direct results linking SG and neurodiversity, separate searches were performed to analyze the relationship between SG and EF, as well as SG and neurodiverse populations. Two independent reviewers assessed the quality and risk of bias of the included studies using the Risk of Bias 2 tool for randomized studies and the Risk of Bias in Non-Randomized Studies of Interventions tool for nonrandomized studies.

**Results:**

The review identified 16 studies that met the inclusion criteria. Of these, 15 addressed the use of SG for improving EF in neurodiverse populations, such as children with attention-deficit/hyperactivity disorder, autism spectrum disorder, and down syndrome. These studies demonstrated significant improvements in various EF domains, including attention, working memory, and cognitive flexibility. However, there was notable heterogeneity in sample sizes, participant ages, and game types. Three studies specifically focused on individuals with down syndrome, showing promising results in improving cognitive functions.

**Conclusions:**

SG hold considerable potential as therapeutic tools for enhancing EF across neurodiverse populations. They have shown positive effects in improving cognitive skills and promoting inclusion in both educational and therapeutic settings. However, further research is required to optimize game design, assess long-term outcomes, and address the variability in study quality. The exclusive inclusion of open access studies may have limited the scope of the review, and future research should incorporate a broader range of studies to provide a more comprehensive understanding of SG’s impact on neurodiversity.

**Trial Registration:**

PROSPERO CRD42024563231; https://tinyurl.com/ycxdymyb

## Introduction

### Background

Neurodiversity is a concept that articulates the inherent variability in human neurological development [1]. It acknowledges the diverse ways in which individuals think, learn, and process information, emphasizing the importance of respecting and valuing these differences [2]. The neurodiversity approach is centered on strengths, aiming to comprehend and support individuals with neurological distinctions, such as autism spectrum disorder (ASD), attention-deficit/hyperactivity disorder (ADHD), and down syndrome (DS) [3,4]. Promoting neurodiversity necessitates cultural shifts within organizations and can guide the efforts of developmental researchers [5].

Neurodiversity or the neurodiverse condition (NC) serves to acknowledge and celebrate the inherent variability within the spectrum of human neurological development [1]. It recognizes that there is not a singular “correct” functioning for the brain; rather, people exhibit a broad spectrum of ways of perceiving and interacting with the world, and these differences deserve respect and encouragement [6]. Coined in the 1990s to combat stigma against individuals with ASD, ADHD, DS and learning disorders like dyslexia, the term has evolved to encompass a wider group of individuals who self-identify as neurodiverse [4]. The associated approach underscores the creation of an inclusive environment that meets the diverse needs of all individuals, steering away from attempts to “fix” or “cure” them [2].

One of the most studied cases of neurodiversity is DS [7-9]. This genetic disorder, the most common cause of cognitive disability, manifests in various phenotypes, including congenital heart defects and Alzheimer disease, impacting individuals to varying degrees [10-12]. Viewing DS through the lens of neurodiversity allows us to recognize and celebrate the unique strengths of everyone, fostering a more inclusive understanding of neurological differences and emphasizing the importance of early interventions and appropriate medical care for improved long-term outcomes [9,13,14].

Children diagnosed with DS may face additional related health problems [15]. For example, it is common for a child with DS to have low muscle tone that requires additional assistance for gross motor developmental milestones such as crawling and walking [5]. There are 3 types of DS: Regular Trisomy 21, Mosaicism, and Translocation [16]. Regular Trisomy 21 is the most usual form and occurs when all cells have an extra copy of chromosome 21 [16]. Mosaicism is a rare form where only some cells have an extra copy of chromosome 21 [16]. Translocation occurs when part of chromosome 21 attaches to another chromosome [17].

Another usual NC is ASD [9,18]. Many children are diagnosed with ASD, a condition that affects how they interact with others, communicate, and learn [19]. Symptoms often appear in the early years of life and can include challenges with social communication and interaction, repetitive behaviors, focused interests, and sensitivities to sensory input [5,6]. Understanding emotions through facial expressions is a crucial part of social interaction, and children with ASD may struggle with this skill [20]. ASD exists on a spectrum, meaning the severity and specific challenges can vary greatly between children [20]. Some children with ASD may be nonverbal, while others may have strong verbal skills but struggle with social cues [20].

On the other hand, many children struggle with ADHD, one of the most common mental health conditions in childhood [21]. Symptoms include inattention (difficulty focusing), hyperactivity (excessive movement that disrupts the environment), and impulsivity (acting hastily without thinking) [22]. ADHD can be chronic and significantly impact a child’s life, affecting academic achievement, friendships, and daily routines [22]. Untreated ADHD can lead to low self-esteem and difficulties interacting with others [23]. This is particularly concerning because ADHD often first appears during school years, when social skills and academic success are crucial [23,24]. While ADHD is more commonly diagnosed in boys, it can affect children of all genders [22].

Other common NC are shown below:

1. Dyslexia: this is a learning disability that affects reading fluency and comprehension [25].
2. Dyspraxia (Developmental Coordination Disorder): this condition affects motor coordination and planning skills [26,27].
3. Dyscalculia: this is a learning disability that affects math skills [28,29].
4. Tourette Syndrome: this condition is characterized by involuntary tics (movements or sounds) [30,31].
5. Synesthesia: this is a neurological condition where stimulation in 1 sense (eg, hearing) leads to a perception in another sense (eg, seeing colors) [32,33].

### Traditional Intervention in Individuals With Neurodiverse Conditions

Children with different NC may share some common characteristics [9]. However, their strengths, challenges, and responses to interventions are unique [1]. There’s no “one-size-fits-all” approach, effective intervention plans must be personalized to each child’s needs and family situation [1]. Some families require more support than others [4]. Intervention strategies for children with NC must be closely linked to assessments of their individual needs and their family’s circumstances [3]. This ensures the intervention can be tailored to maximize the child’s strengths and address specific challenges faced by both the child and the family [4].

The typical sequence of developmental skill acquisition for neurodiverse children may be slower for children with specific NC, compared with typical developing children [9]. This can manifest in delays in learning skills like sitting, standing, walking, or speaking compared with their peers. In addition, these children may require more repetition and consistent practice to learn new skills [1,4].

Due to frequent language delays in children with NC as dyslexia, DS or ASD, early intervention to improve communication is crucial [9]. Ideally, it should begin shortly after birth and be integrated into their daily activities, making it an ongoing process [3]. Collaboration with a speech-language pathologist can provide consistent intervention for both the child and their parents [3]. The focus should be on stimulating vocalization, developing receptive and expressive language [3].

Motor development encompasses learning to perform actions like sitting, standing, manipulating objects, caring for oneself, and engaging in play or work activities. This process relies on the brain’s ability to process sensory information and translate it into purposeful movements [34]. Gross motor skills involve larger muscle groups (neck, trunk, and limbs), while fine motor skills involve precise use of hands and fingers [25]. Early intervention in motor development is essential for children with NC as dyspraxia or DS [25].

It is recommended that interventions for motor function use techniques that [35] use diverse types of stimuli to generate movement, depending on the child’s response, assist the child in performing movements they are capable of but may not know how to do independently, and support the child in problem-solving and participating in the planning, initiation, and execution of movements.

Interventions targeting social development focus on social attention, social interactions, bonding, and play [16,19]. These interventions help parents interact with their children, which is especially important for children with DS as they often show less initiative, react, and interact in more unpredictable ways, express fewer emotions, and exhibit difficulties in their social and communicative skills, making their interpretation challenging [15,16].

There are various intervention approaches to consider for a child with NC, including both traditional and nonconventional therapies [3,4,9]. These approaches may vary in terms of application, time, cost, and potential benefits or risks. Some therapies incorporating sensory activities may be beneficial for the child’s overall development [1]. Generally, it is important for these therapies to be implemented by providers with knowledge and experience in the field [1,3].

### Executive Functions

Executive function (EF) is a general term used to describe the cognitive processes necessary for regulating and controlling adaptive and goal-directed behavior, which includes skills such as working memory (WM; ie, short-term memory), the ability to inhibit or stop specific actions, task switching, and planning [36-38]. Decision-making as an executive function involves reasoning processes to generate heuristics for intuitive or analytical answers based on experience [36-38]. Verbal fluency, an executive function task, involves the ability to generate, produce, express, and relate words, encompassing both semantic and phonemic fluency [36-38]. These skills can be divided into 2 categories: “cool” and “hot” skills. “Cool” skills are knowledge-based cognitive processes, such as working memory and planning [39], while “hot” skills are related to emotional regulation and motivation, such as the ability to inhibit behaviors and control emotions [39,40]. Together, EF skills allow individuals to manage complex cognitive processes [41] and are important for daily functioning, social interactions, and academic success [18,42]. The unity and diversity of these skills highlight the interconnected yet distinct nature of different EF components [43,44].

Cognition encompasses the brain processes that allow us to remember, think, act, experience emotions, and perceive our environment [45]. Cognitive processes are diverse, complex, and interrelated in significant ways [42]. Key aspects of cognition include attention and exploration, learning and memory, as well as the ability to reason and solve problem [19,45]. Individuals with NC often exhibit weaknesses in various EF, such as planning, organization, monitoring progress, and work quality, as well as impulse control [45]. The interrelation of these cognitive processes is supported by the framework of unity and diversity within EF, where individual differences offer insight into the broader cognitive structure [43,44]. Fortunately, the frontal lobes, where these functions largely reside, continue to develop in young adults, and improvement in EF has been observed in many individuals with NC who receive different kind of therapy [19,46]. Impulsive behaviors, such as running or hitting, are likely to improve with age. However, in young children, it is often necessary to provide support to enhance their EF, for example, through the use of reminders to stay on task or breaking tasks into smaller steps to assist with planning and organization and providing reminders to control their impulses [19,45].

Therefore, it is crucial to identify strengths and weaknesses in EF in order to understand the developmental pattern of individuals with NC throughout their lives, ultimately helping them achieve success in cognition, academic ability, and social activity [46]. Several studies on EF in individuals with DS have focused on children and adolescents, using rating scales such as the Behavior Rating Inventory of Executive Function (BRIEF) [46] and the BRIEF-Preschool Version (BRIEF-P) [47], which are parent or teacher-rated and designed to assess multiple aspects of EF in children aged 6-18 years and 2-5 years, respectively [46]. These scales include clinical subscales (eg, working memory), broad indexes (eg, metacognition), and an overall composite score (Table 1).

**Table 1:** Components and descriptions of the BRIEF^a^ and BRIEF-P^b^ scales [46-49].

Scale Name		Description	
Inhibition		Ability to interrupt or control behavior at the appropriate time	
Change		Ability to move freely from one situation, activity, and aspect of a problem to another	
Emotional control		Ability to modulate emotional responses	
Working memory		Ability to retain information in mind in order to complete a task or provide a response	
Planning and organization		Ability to manage demands of a current or future-oriented task within a specific situational context	
**Other subscales in the BRIEF**			
	Initiation		Ability to generate ideas, answers, and strategies to solve problems
	Organization of materials		Proceed methodically in work, play’ and save space in cabinets and drawers
	Monitoring		Habits of checking work and personal follow-through
**Broad indexes in BRIEF-P**			
	Index of self-control to inhibit		Capacity for inhibition + emotional control
	Emergent metacognition index		Working memory + planning or organization
	Flexibility index		Capacity for change + emotional control
**Broad indexes in BRIEF**			
	Behavior regulation index		Capacity for inhibition + for change + emotional control
	Metacognition index		Ability to initiate + working memory + planning or organization + organization of materials + follow-through
**BRIEF and BRIEF-P global composite**			
	Executive global composite		The set of executive functions–summary of all clinical subscales

^a^BRIEF: Behavior Rating Inventory of Executive Function.

^b^BRIEF-P: BRIEF-Preschool Version.

One of the main discrepancies in the brain of individuals with NC is found in the areas of learning and memory [13]. The hippocampus and the temporal lobe, which are essential for the acquisition and retention of new information, show notable differences in some NC as DS [13]. When a typically developing child learns something new, their brain processes the information, retains it in short-term memory, encodes it, and then stores it in long-term memory [49]. However, in the case of children with DS, the transfer of information to long-term memory and its storage is not as straightforward or consistent [49]. This may explain why these children often learn better through repetition and review of concepts or tasks, rather than a single exposure to the information [11]. Consequently, the fact that a child with DS sees or hears something only once may not allow them to retain the information [11].

### Serious Games

Serious games (SGs) are games designed to achieve a specific objective beyond entertainment, such as learning, training, problem-solving, or decision-making [16,28]. These games combine playful elements with educational or training elements, making them useful in a wide variety of fields, including education, health care, defense, business, government, and skills training [21,23,50,51].

These games are designed for a formative or educational purpose, rather than solely for entertainment purposes [16,48]. They focus on the specific design of the learning process by creating scenarios that allow players to learn and practice specific skills [15]. SG are a combination of methods and technologies that educate and train players to change their behaviors [16]. In education, SG are used to foster students’ emotional intelligence and raise awareness of relevant social issues. In skills training, they are used for learning mathematics, languages, and programming, among others [15].

In health care, SG are used by clinicians for patient rehabilitation and different kinds of therapies [16]. They can be used to improve treatment adherence, health education, and disease prevention [28]. For example, SG has been developed for the rehabilitation of patients with brain injuries, the treatment of mental disorders, and education on healthy habits [16]. SG can be an innovative alternative to improve the quality of health care and the patient experience [31].

SG can take different forms and formats, from straightforward digital games to advanced simulations utilizing 3D environments [50,51]. These games often use gaming techniques such as immediate feedback, competition, goal achievement, and collaboration to keep players interested and engaged [25,32,33]. They are an effective form of interactive learning that combines fun and education to achieve a specific outcome [29-31,52].

SG have proven to be a useful tool for intervening in EF in children with NC [16,20,22,24,28]. These games can help develop executive skills in a playful and enjoyable context, as well as teach various academic subjects [51,53]. Consequently, the aim of this review article is to explore and analyze 3 topics that have received significant attention in recent research: NC, EF, and SG, focusing on how the latter has positively intervened in children with NC. Knowing this context of variability and unique needs within the spectrum of neurodiversity, the following research question was posed: What are the effects of using serious games on the executive functions of children with neurodiversity?

## Methods

### Overview

To conduct this systematic review, the methodological guidelines of PRISMA (Preferred Reporting Items for Systematic Reviews and Meta-Analyses) [54] were followed. This approach ensures a rigorous and transparent process in the search, selection, critical appraisal, and synthesis of relevant studies. The following PICOS (Population, Intervention, Comparison, Outcome, Study design) framework was used to define the inclusion and exclusion criteria for the study as follows:

1. Population: the study focuses on individuals with cognitive disorders, encompassing neurodiverse conditions.
2. Intervention: the study evaluates the use of serious games as an intervention tool to enhance executive functions in this population.
3. Comparison: comparisons may involve no intervention, traditional nonserious game-based interventions, or other forms of nongame interventions.
4. Outcome: the objective is to measure the impact of serious games on the development of executive functions in individuals with neurodiversity.
5. Study design: methods consistent with a systematic review were used, following the methodological guidelines of PRISMA. These included a systematic literature search, critical appraisal of included studies, and synthesis of relevant findings.

### Search Strategy

The search was performed using the Boolean method, including the “AND/OR” operators only for studies that contained relevant key terms. Original research articles that identified the SG and neurodiversity and SG and EF were analyzed. The research was performed on June 20, 2024, in 3 databases (Scopus, Web of Science, and PubMed) using the next 2 search equations:

- (“serious games” OR “digital games”) AND (neurodiversity)
- (“serious games” OR “digital games”) AND (“executive functions”)

We opted to perform 2 search equations due to the lack of results when attempting to combine the 3 main themes of the research (serious games, executive functions, and neurodiversity). This separation allowed for a more specific and focused search on each of these aspects, facilitating the identification of relevant studies for the systematic review.

From 2019 to the present, only articles published in open access journals, all open access and journal type. Review articles were not included. Furthermore, we only included studies that used SG for children with neurodiversity or typical development (TD) emphasizing the impact of development of EF. After the exclusion of duplicates, the title and abstracts were read, with nonrelevant articles excluded by age, document type, and study type. The articles were selected after complete reading according to the inclusion and exclusion criteria. Two reviewers independently conducted their analysis using Rayyan, a web and mobile app designed for systematic reviews [55], to mitigate the risk of bias in article evaluations. This approach facilitated the assessment of study quality based on parameters such as randomization and blinding. In instances where discrepancies arose regarding study quality, a third reviewer was consulted for resolution.

### Inclusion and Exclusion Procedures

For each search equation, the results were assessed against the following inclusion criteria: first, the publication date of the documents had to be within the last 5 years, specifically from 2019 to the present year. In addition, only research articles were considered eligible for inclusion. These criteria are summarized in Textbox 1.

**Inclusion and exclusion criteria for systematic review.**

**Inclusion criteria**

- Open access: all open access.
- Publication year: 2019 to June 20, 2024.
- Document type: article.
- Source type: journal.
- Age: including < 18 years.
- Study Type: original research articles using serious games for neurodiversity or typical development.

**Exclusion criteria**

- Open Access: not all open access.
- Publication year: < 2019.
- Document type: reviews, conference papers, letters, and books.
- Source type: nonjournal sources.
- Age: ≥ 18 years.
- Study type: articles that did not specify the use of serious games.

### Quality Assessment

During the quality assessment process, 2 objectives were established to ensure the rigor and relevance of the studies included in this systematic review. First, the focus was on specificity in the research topic, ensuring that selected articles were clearly centered on the use of SG as an intervention tool to enhance EF in individuals with neurodiversity. This led to the exclusion of studies that did not adequately specify the use of serious games in this context. Second, depth of analysis was prioritized by selecting studies that provided a detailed and specific analysis of the impact of SG on EF in individuals with neurodiversity, excluding those that only offered a general overview of the topic without focusing on specific outcomes. These criteria ensured methodological coherence and relevance of the studies included for the objectives of this systematic review.

For this literature review, we used the RoB2 [56] and ROBINS-I [57] scales to systematically assess the quality and validity of the studies included. Randomized studies were evaluated using the RoB2 scale, while nonrandomized studies were evaluated with the ROBINS-I scale. In addition, we used the ROBVIS tool [58] to create risk-of-bias plots, which helped visualize the bias assessments for each study. This comprehensive approach allowed us to identify studies with a high risk of bias, providing a more rigorous and reliable interpretation of the aggregated results in our review.

### Data Extraction

A brief description of the relevant data extracted from the included studies in the systematic review is presented in Textbox 2.

**Brief description of relevant data extracted from studies included in the systematic review.**

**Title**

- The title of the study or intervention.

**Reference**

- The bibliographic reference of the study, including authors and year of publication.

**Year**

- The year in which the study was conducted.

**Sample size**

- The size of the sample, i.e., the number of participants included in the study.

**Age**

- The age of the participants in the study.

**Health condition**

- The specific health condition addressed by the study, for example, Down syndrome or other neurodiverse conditions.

**Intervention**

- The intervention or treatment applied to the participants.

**Serious game name**

- The name of the serious game used as part of the intervention.

**Objective of the game**

- The specific objective of the serious game in the context of the study.

**Technology**

- The technology used to implement serious games, such as computers, tablets, mobile devices, etc.

**Software used**

- The specific software used to run the serious game.

**Supervision**

- The level of supervision provided during the intervention, which may range from direct supervision to autonomous.

**Sessions**

- The number of sessions in which the intervention was administered.

**Duration (min)**

- The duration of each intervention session, measured in minutes.

**Frequency (times/week)**

- The frequency at which the intervention was administered, expressed as the number of times per week.

**Period (month)**

- The total time over which the intervention was conducted, expressed in months.

**Executive Functions**

- The specific assessment or measure of executive functions used in the study.

**Experimental design**

- The experimental design used in the study, which may include randomized controlled trials, longitudinal studies, etc.

**Validation**

- Information about the validation of the study or the assessment tool used, such as construct validity or criterion validity.

The variables analyzed were extracted in the results. In case of missing data, they were reported as NR (Not reported).

## Results

**Figure 1:**
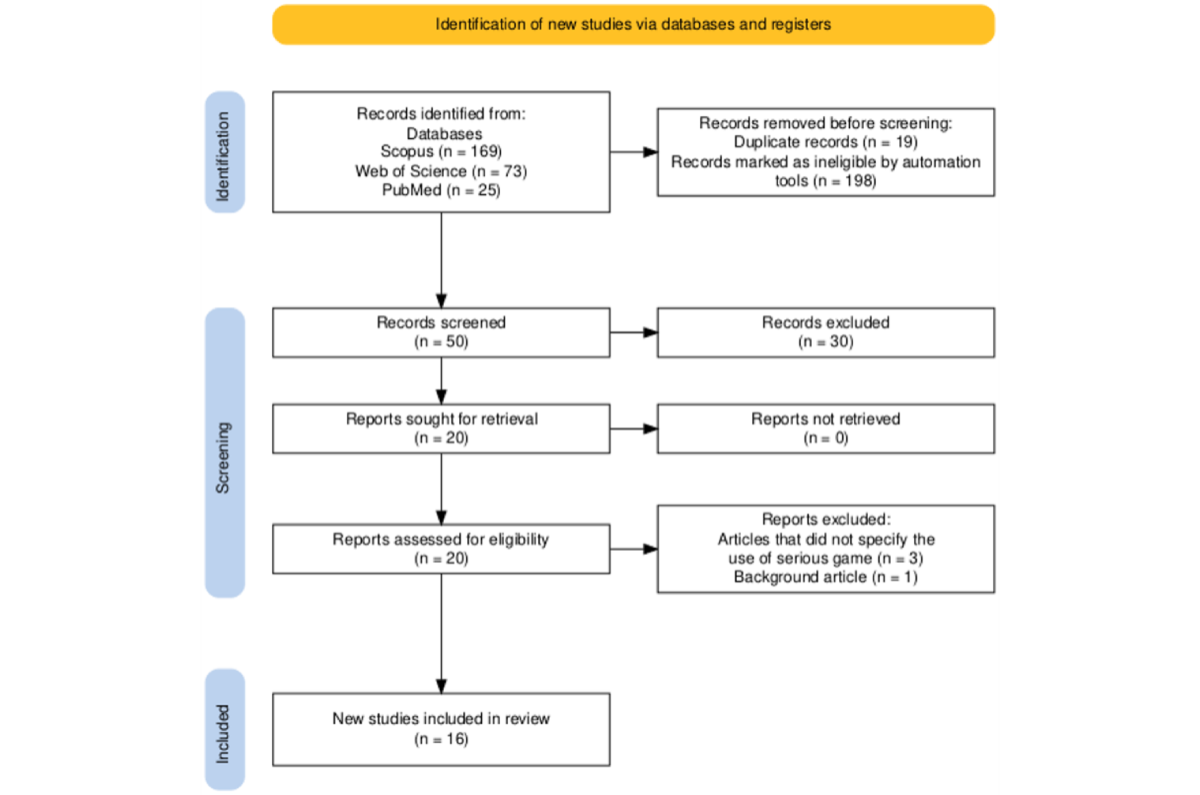
Preferred Reporting Items for Systematic Reviews and Meta-Analyses flowchart for identification of studies by databases.

The initial search included 267 articles from Scopus, Web of Science, and PubMed. After removing duplicate studies and applying the search filters included in the inclusion criteria, 50 potentially relevant articles were identified. Subsequently, 30 studies were excluded based on the screening of titles, keywords, and abstracts. The eligibility of the remaining 20 full-text articles was assessed, resulting in the exclusion of 4 articles for distinct reasons, such as the lack of specification regarding the use of serious games [59-61] and articles that provide only an overview [62]. Consequently, 16 articles were included in the final analysis. Figure 1 presents a flowchart detailing the identification and selection process of the studies.

Multimedia Appendix 1 and Table 2 offer a comprehensive overview of the extracted data from the included studies [21-32,50,63-66], following the PRISMA methodology. The appendix summarizes the relevant data, while Table 2 provides a detailed analysis of key variables such as intervention, sessions, duration (min), frequency (times/week), period (month), executive functions, and experimental design. This structured presentation allows for a direct comparison of methodological approaches across studies. Understanding how specific characteristics, as well as the volume and intensity of interventions, influenced improvements in executive functions across different neurodiversities is crucial. This enriched discussion on intervention effectiveness clarifies current findings and lays a solid foundation for future research in the field.

**Table 2:** Summary of experimental designs, intervention details, and outcomes across studies.

No.	Intervention	Sessions	Duration (min)	Frequency (times/week)	Period (month)	Executive functions	Experimental design
1	Serious game and pencil and paper format	16	30-45	2	2	Regulating attention, inhibitory control	Yes
2	Serious game and commercial cognitive training system	1	90	1	<1	Regulating attention, inhibitory control, Working memory	Yes
3	Serious game	24	NR^a^	2	3	Regulating attention, working memory, problem-solving	No
4	Serious game	NR^a^	180	NR^a^	3	Regulating attention, planning and organization , self-regulation	Yes
5	Exergame	25	20	3	3	Inhibitory control	Yes
6	Prototype game and placebo version of the game	NR^a^	NR^a^	NR^a^	NR^a^	Shifting task	Yes
7	Serious game and conventional therapy	NR^a^	50	1	6	Regulating, planning and organization, working memory, meta cognition, shifting task, self-regulation, inhibitory control, problem-solving, cognitive flexibility	Yes
8	Serious game and therapeutic chess. control group	12	NR^a^	1	3	Planning and organization, working memory, self-regulation, inhibitory control, shifting task, cognitive flexibility	Yes
9	Serious game	3	45	NR^a^	NR^a^	Working memory, shifting task, inhibitory control	Yes
10	Serious game	1	10	NR^a^	NR^a^	Regulating attention, planning and organization, working memory, inhibitory control, cognitive flexibility	Yes
11	Serious game	1	90	1	NR^a^	Regulating attention, planning and organization, working memory, inhibitory control, cognitive flexibility	No
12	Serious game and no serious game	15	15	3	<2	Inhibitory control	Yes
13	Serious game and mathematical video game	12	20	2	<2	Inhibitory control, shifting task	Yes
14	Serious game	50	NR^a^	NR^a^	NR^a^	Inhibitory control, cognitive flexibility, working memory	Yes
15	Serious game and passive intervention	12	15	2	<2	Regulating attention, inhibitory control, working memory, cognitive flexibility, planning and organization	Yes
16	Adaptive cognitive training program and commercial program	25	20	5	<2	Regulating attention, inhibitory control, working memory	Yes

^a^NR: not reported.

### Analysis of Included Studies With Implementation of Experimental Design

Gallardo and Vergara [24] conducted a 2-factor ANOVA with repeated measures, comparing 2 groups (experimental with SG and control with traditional treatment) evaluated at 3 points (start, ninth session, and end of treatment). The study included children with ADHD in both groups. With 16 sessions of 30-45 minutes, conducted twice weekly over 2 months, the researchers observed significant improvements in selective and maintained attention and a greater reduction in impulsivity within the experimental group.

Menestrina et al [50] compared an intervention with SG with a control group using BrainHQ, with pre and postintervention evaluations. Sessions lasted 60 minutes, and the number of sessions correlated with improvements. Notable improvements were found in attention and working memory, with variations depending on neurodiversities such as ADHD and dyslexia, leading to a recommendation for personalized interventions.

Coma-Rosellé et al [22] used participant observation of 27 children with ADHD, recording field notes and holding interdisciplinary discussion groups. Sessions lasted 10-15 minutes over 3 months, resulting in improvements in planning and attention with educator mediation. However, a more detailed analysis is necessary to establish a clear relationship.

Mossmann et al [32] conducted a pilot study with 7 students with TD using an exergame over 3 months, playing 1528 rounds, and using exploratory analysis and logistic regression. The study found performance differences based on difficulty levels and a positive correlation between performance and difficulty.

Robb et al [26] performed a randomized controlled trial with 2 groups (active training and placebo), totaling 2 hours and 45 minutes in active training sessions. They observed improvements in task switching in children with Prader-Willi syndrome, with less improvement seen in the placebo group.

Schena et al [23] executed a randomized controlled trial involving 60 children with ADHD, using interventions with virtual reality and traditional therapies. Although specific details were not provided, the study highlighted the need for further analysis to determine the influence of volume and intensity on different neurodiversities.

Rodrigo-Yanguas et al [21] conducted a randomized controlled trial using a chess-based video game over 12 training sessions, with 1 session per week divided into 3 blocks. They reported improvements in emotional control and regulation of the ADHD population analyzed but additional studies were recommended.

Crepaldi et al [27] used a quasi-experimental design with sessions involving a serious game and standardized tests in TD children. Although no significant differences were found in serious game scores, there were positive correlations between impulsivity scores, indicating the need for further research to determine effectiveness.

Sanchez-Morales et al [29] applied contextual rapid design techniques and participatory design without providing specific data in TD children. They recommended including specific data and correlational analysis to better understand the influence of the interventions.

Ramos and Garcia [31] conducted a quasi-experimental study with participant and control groups of TD children, using pre- and postintervention evaluations. Sessions lasted 15 minutes, 3 times per week, over 5 weeks, leading to significant improvements in inhibitory control, attention, and short-term memory, with a recommendation for personalized interventions.

Lê et al [25] compared a digital game for fine motor skills training with a math game in a controlled experimental design. Although specific data were not provided, the intervention lasted approximately 4 hours over 6 weeks, with TD children. Improvements in motor and literacy skills were observed, with a recommendation to include data on volume and intensity to assess impact on EF across different neurodiversities.

Eng et al [64] used a within-subjects design to compare the traditional Flanker Task with the gamified “Frankie’s Big Adventure” in TD children. Specific data were not provided, and future studies were suggested to address these issues, emphasizing the need to evaluate the influence of volume and intensity on the improvement of executive functions.

Feria-Madueño et al [65] conducted a randomized controlled trial comparing active intervention (video game) with passive intervention (video viewing). Over 6 weeks, with 3 sessions per week, each lasting approximately 120 minutes and including 15 minutes of specific training, significant improvement in the video game success rate was observed in TD children. The study recommended including data on neurodiversities to evaluate effectiveness across different profiles.

Richmond et al [66] performed a randomized controlled trial comparing digital cognitive training with a control group of ADHD children. The intervention consisted of 10 sessions (2 per week over 5 weeks), each lasting 30 minutes. They expected a positive correlation between greater volume and intensity and improvements in executive functions, with an analysis needed to optimize interventions for different neurodiversities.

### Risk of Bias

**Figure 2:**
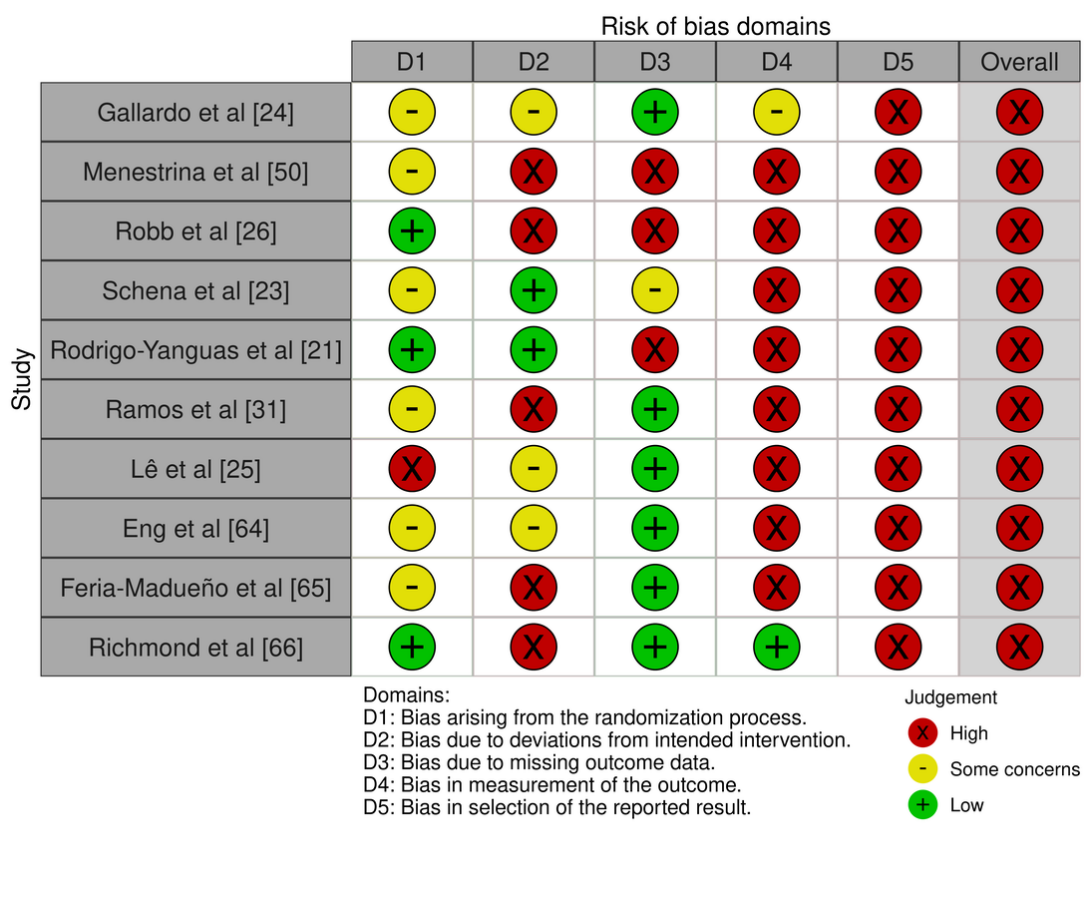
Tabular representation of risk of bias in individual studies evaluated using Risk of Bias 2 tool [21,23,24-26,31,50,64-66].

Figure 2 [21,23-26,31,50,64-66] shows the risk of bias assessment in various studies using the RoB2 tool, which consists of 5 main domains (D1 to D5). The evaluated domains include bias arising from the randomization process (D1), bias due to deviations from intended interventions (D2), bias due to missing outcome data (D3), bias in the measurement of the outcome (D4), and bias in the selection of the reported result (D5). The overall risk of bias assessment is also presented for each study.

Most of the evaluated studies present a high risk of bias overall. Specifically:

- Gallardo and Vergara [24]: shows “some concerns” in domains D1, D2, and D4, and a high risk in D5, resulting in a high overall risk of bias.
- Menestrina et al [50]: displays a high risk in domains D2, D3, D4, and D5, with “some concerns” in D1, leading to a high overall risk.
- Robb et al [26]: presents a low risk in D1 but a high risk in the remaining domains (D2 to D5), resulting in a high overall risk.
- Schena et al [23]: has “some concerns” in D1 and D3, and a high risk in D4 and D5, leading to a high overall risk.
- Rodrigo-Yanguas et al [21]: shows a low risk in D1 and D2, but a high risk in D3, D4, and D5, resulting in a high overall risk.
- Ramos and Garcia [31]: presents “some concerns” in D1 and D3, and a high risk in D2, D4, and D5, leading to a high overall risk.
- Lê et al [25]: has a high risk in D1, D4, and D5, and “some concerns” in D2, resulting in a high overall risk.
- Eng et al [64]: presents “some concerns” in D1, D2, and D3, and a high risk in D4 and D5, leading to a high overall risk.
- Feria-Madueño et al [65]: shows “some concerns” in D1, a high risk in D2, D4, and D5, and a low risk in D3, resulting in a high overall risk.
- Richmond et al [66]: presents a low risk in D1, D3, and D4, and a high risk in D2 and D5, leading to a high overall risk.

**Figure 3:**
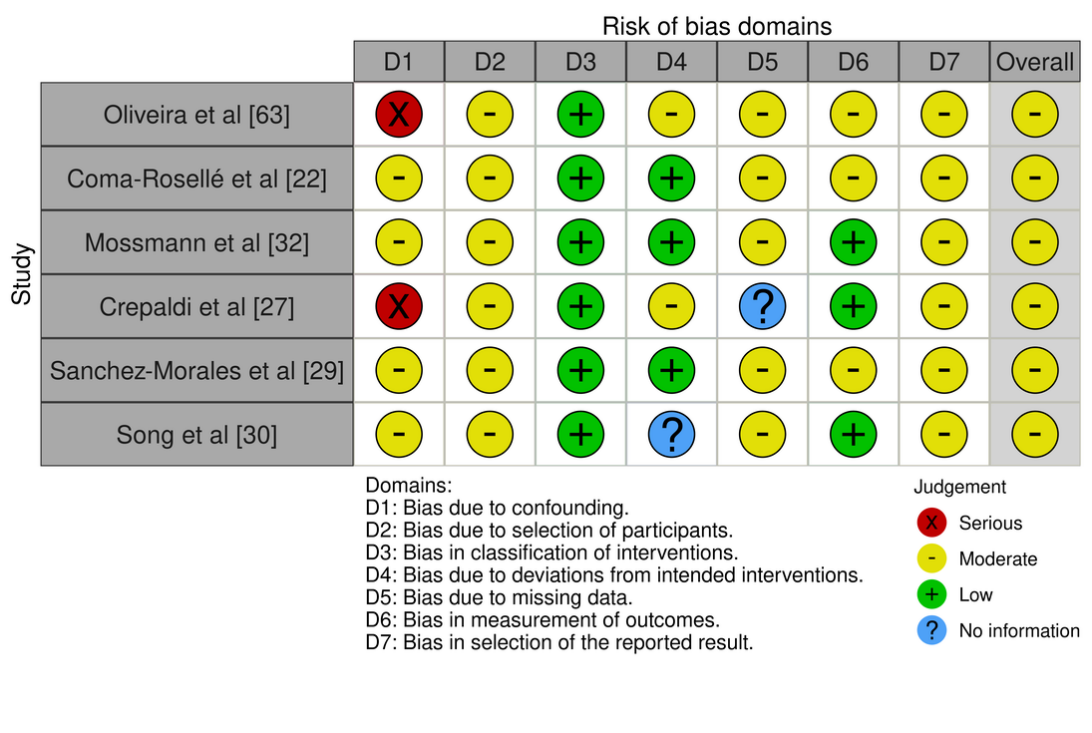
Tabular representation of risk of bias in individual studies evaluated using Risk of Bias in Non-Randomized Studies of Interventions-I tool [22,27,29,30,32,63].

Figure 3 [22,27,29,30,32,63] shows the risk of bias assessment in various studies using the ROBINS-I tool, which evaluates nonrandomized studies. The evaluated domains (D1 to D7) include:

1. D1: Bias due to confounding.
2. D2: Bias in the selection of participants.
3. D3: Bias in the classification of interventions.
4. D4: Bias due to deviations from intended interventions.
5. D5: Bias due to missing data.
6. D6: Bias in the measurement of outcomes.
7. D7: Bias in the selection of the reported result.

Each domain is evaluated with categories including “Serious,” “Moderate,” “Low,” and “No information.” The overall risk of bias assessment is also presented for each study.

- Oliveira and Ramos [63]: this study has a serious risk in D1 and a moderate risk in D2, D4, D5, D6, and D7, with a low risk in D3. The overall assessment is moderate.
- Coma-Rosellé et al [22]: shows a moderate risk in D1, D2, D5, D6, and D7, and a low risk in D3 and D4, resulting in a moderate overall assessment.
- Mossmann et al [32]: has a moderate risk in D1, D2, D5, and D7, and a low risk in D3, D4, and D6. The overall assessment is moderate.
- Crepaldi et al [27]: presents a serious risk in D1, a moderate risk in D2, D4, and D7, and a low risk in D3 and D6, with no information in D5. The overall assessment is moderate.
- Sanchez-Morales et al [29]: shows a moderate risk in D1, D2, D5, D6, and D7, and a low risk in D3 and D4. The overall assessment is moderate.
- Song et al [30]: has a moderate risk in D1, D2, D5, and D7, and a low risk in D3 and D6, with no information in D4. The overall assessment is moderate.

## Discussion

### Principal Findings

This systematic review reveals that SG have a significant impact on enhancing various executive functions across diverse health conditions and age groups. The findings indicate that SG can effectively improve attention, working memory, cognitive flexibility, and inhibitory control. For instance, SG interventions such as those studied by Gallardo and Vergara [24] and Schena et al [23] demonstrated notable improvements in attention and executive functions among children with ADHD. In addition, studies like those of Feria-Madueño et al [65] and Lê et al [25] illustrate the benefits of SG in sports performance and literacy development.

### Improvement of Cognitive Skills

The reviewed studies consistently show that SG can enhance various EF across different health conditions and age groups. For example, Gallardo and Vergara [24] found significant improvements in attention and inhibitory control in children with ADHD. Similarly, Schena et al [23] demonstrated improvements in multiple EF, including working memory and cognitive flexibility, through cognitive-behavioral training. In addition, Feria-Madueño et al [65] indicated improvements in attention processes and sports performance in young soccer players.

### Inclusion in Educational Settings

SG have been successfully integrated into educational settings for children with diverse cognitive needs. Menestrina et al [50] and Oliveira and Ramos [63] highlight how these games can assist in the education of children with dyslexia and typical development, respectively. In addition, games like “Apollo and Rosetta” [32] and “Play with SID” [29] have been designed for typically developing children to help identify and improve cognitive deficiencies. Lê et al [25] demonstrates how digital games can be used to train fine motor skills and improve literacy development. Further investigation is required to explore how SG can enhance the participation of neurodiverse individuals in learning and educational spaces, fostering a more inclusive environment.

### Promising Therapeutic Tools

The variety of technologies used (PC, tablets, smartphones, and VR) suggests that SG are promising therapeutic tools. This is evident in studies such as those written by Coma-Rosellé et al [22] and by Feria-Madueño et al [65]. Games like “IAmHero” [23] show the potential of VR to enhance executive functions in children with ADHD. In addition, Crepaldi et al [27] highlights how serious games can be used to evaluate inhibition mechanisms in children, demonstrating their utility in both assessment and intervention.

### Variability in Sample Sizes and Participant Ages

The included studies show considerable variability in sample sizes and participant ages:

1. Sample sizes: sample sizes range from small-scale studies with 5 participants, as seen in the study by Robb et al [26], to large-scale investigations with hundreds of subjects, such as Menestrina et al [50].
2. Age range: the studies cover a wide age spectrum, from children as young as 3 years Coma-Rosellé et al [22] and 5 years Eng et al [64] to young adults up to 22 years Rodrigo-Yanguas et al [21], reflecting the applicability of SG across different developmental stages.

### Technologies Used

The variety of technological platforms used in SG highlights their versatility and accessibility:

1. Smartphone and tablet games: representing a significant portion of the studies (eg, Coma-Rosellé et al [22] and Ramos and Garcia [31]), these platforms demonstrate their popularity and ease of access. Similarly, the assessment by Song et al [30] was developed in unity for smartphone use for cognitive control.
2. Computer games: used in several studies (eg, Oliveira et al [63], Schena et al [23]), highlighting their versatility in therapeutic interventions.
3. Virtual reality: although less common (eg, Schena et al [23]), VR offers an immersive experience that can be particularly beneficial in certain therapies.
4. Additional technologies: elements like cameras and sensors were used in some studies to enhance the gaming experience and provide more precise data for therapeutic purposes, as in Sanchez-Morales et al [29].

### Supervision and Session Design

The supervision and design of gaming sessions also varied considerably:

1. Supervision: SG were supervised by health professionals, researchers, or parents, providing both close supervision and autonomy to the participants.
2. Session design: the duration, frequency, and number of sessions varied depending on the study design and intervention goals. Studies ranged from weeks to months, allowing for the analysis of the potential long-term effects of SG interventions on executive functions.

### Limitations and Future Needs

While the findings are promising, there are limitations that need to be addressed:

1. Lack of long-term validation: many studies lack long-term follow-up, limiting the understanding of the sustained effects of SG interventions.
2. Experimental design: some studies did not use robust experimental designs, which could affect the validity of the results.
3. Game design optimization: more research is needed to optimize game design elements and assess their impact on different aspects of neurodiversity. Therefore, serious games have great potential to improve cognitive skills and promote the inclusion and participation of people with diverse cognitive profiles in educational settings. Emerging technologies, such as web applications, robotics, and virtual reality, also show promise as therapeutic tools. However, more research is needed to explore long-term effects, optimize game design, and assess their impact on various aspects of neurodiversity.
4. Inclusion of both open access and traditional studies: the focus on open access studies may have excluded relevant research published in traditional formats, particularly from research teams with limited funding for open access fees. Future reviews should consider incorporating both types of studies to provide a more comprehensive overview.
5. Limitation of time frame: the review focused on studies from the past 5 years to capture the most current trends and advancements. However, this approach may exclude significant findings from older studies. This limitation suggests that future reviews should include older studies and conduct sensitivity analyses to compare the stability or changes in results across different periods.
6. Risk of bias: the risk of bias assessment using the RoB2 and ROBINS-I tools revealed several limitations in the included studies. Most studies evaluated with the RoB2 tool presented a high overall risk of bias, particularly due to issues in randomization, deviations from intended interventions, and missing outcome data. Similarly, studies assessed with the ROBINS-I tool exhibited moderate to serious risk of bias, mainly due to confounding factors, selection of participants, and deviations from interventions. These limitations highlight the need for more rigorously designed studies to ensure the reliability and validity of the findings in future research.

### Conclusions

This paper presents a systematic review of SG and their applications in enhancing executive functions within the framework of neurodiversity. The review explores the current landscape and emerging trends in the use of serious games for cognitive training and therapeutic purposes, highlighting advancements in game design and the integration of various technologies. The diverse applications of SG in educational and therapeutic settings are discussed, emphasizing their role in promoting inclusion and supporting individuals with developmental and intellectual disabilities. Through detailed analysis, the paper examines the effectiveness, potential benefits, and challenges associated with SG, underscoring the need for further research to optimize these tools for a broader range of cognitive functions and diverse populations.

The insights gleaned from the reviewed articles on SG and cognitive functions underscore the potential of these innovative approaches within the context of neurodiversity. SG designed specifically to enhance cognitive abilities and foster inclusion in educational settings for individuals with intellectual disabilities show promise. In addition, web applications featuring serious games emerge as valuable adjuncts to therapy for children with intellectual disabilities, promoting increased engagement and potentially yielding improved outcomes while aligning with the principles of neurodiversity.

Furthermore, the integration of robotics and serious games holds promise as a therapeutic tool for children with developmental disorders, offering avenues to enhance enjoyment and motivation during therapy sessions. However, it is crucial to acknowledge the variability in the effectiveness of serious games across specific games and populations, emphasizing the importance of meticulous evaluation and monitoring a perspective aligned with the principles of neurodiversity that recognize diverse cognitive responses.

The application of virtual reality as a tool for cognitive rehabilitation in children with traumatic brain injuries and for augmenting executive functions in children with ADHD further aligns with the inclusive principles of neurodiversity. Game design elements, including difficulty curves and adaptive algorithms, play a crucial role in enhancing cognitive stimulation and skill development among players, reflecting a tailored and diversified approach that resonates with the concept of neurodiversity.

In addition, the potential demonstrated by exergaming, which combines aerobic exercise and gaming, in improving cognitive functions and executive function in individuals with metabolic syndrome, underscores the versatility of serious games in catering to diverse populations. The development and evaluation of serious games for cognitive training in older adults, yielding positive effects on attention, EF, and speech processing capacity, further highlight the potential of these tools across the lifespan within the inclusive paradigm of neurodiversity.

While these findings illuminate the effectiveness of SG for cognitive training, skill development, and rehabilitation, further research is imperative to explore long-term effects, optimize game design, and evaluate specific impacts on different cognitive functions and populations all while embracing the principles of neurodiversity to ensure a holistic and inclusive approach.

### Data Availability

The datasets used or analyzed during the current study are available from the corresponding author on reasonable request. The review has been registered in PROSPERO (CRD42024563231). A URL has been created that is open access and contains the necessary information to work with the article [67].

## Appendix

Multimedia Appendix 1 Main review of studies selected.

Multimedia Appendix 2 PRISMA checklist.

## Abbreviations

ADHD: attention-deficit/hyperactivity disorder
ASD: autism spectrum disorder
BRIEF: Behavior Rating Inventory of Executive Function
BRIEF-P: BRIEF-Preschool Version
DS: down syndrome
EF: executive function
NC: neurodiverse condition
PICOS: Population, Intervention, Comparison, Outcome, Study design
PRISMA: Preferred Reporting Items for Systematic Reviews and Meta-Analyses
ROBINS-I: Risk of Bias in Non-Randomized Studies of Interventions
SG: serious games
TD: typical development
WM: working memory
